# Quantification of the severity of tricuspid regurgitation from cardiac magnetic resonance cine images

**DOI:** 10.1186/1532-429X-18-S1-P343

**Published:** 2016-01-27

**Authors:** Diego Medvedofsky, Javier Leon Jimenez, Karima Addetia, Roberto Lang, Victor Mor-Avi, Amit R Patel

**Affiliations:** 1 grid.170205.10000000419367822Cardiology, University of Chicago, Chicago, IL USA; 2 Cardiology, Jerez de la Frontera Hospital, Cadiz, Spain

## Background

Today, there is no accepted approach to evaluate tricuspid regurgitation (TR) using cardiovascular magnetic resonance (CMR). Contrary to 4-chamber imaging planes, which often do not show the entire regurgitant jet, these jets are readily visualized in short-axis views in the right atrium (RA). We hypothesized that the size and signal intensity (SI) of the cross-sectional jet area in the short-axis views would reflect TR severity.

## Methods

We studied 61 patients with ≥mild TR on echocardiography, who underwent CMR within 24 hours. The severity of TR was determined by color Doppler vena contracta (VC): severe (VC≥7 mm; N = 20), moderate (3<VC<7 mm; N = 21) and mild (VC≤3 mm; N = 20). CMR TR jet area and mean SI (normalized by that in the RA cavity away from the jet) were measured in a single short-axis frame that depicted maximum area. Receiver-operating characteristic (ROC) analysis was performed on a subgroup of 21 patients for each parameter to determine its diagnostic accuracy for differentiating degrees of TR and the optimal cutoffs, which were then independently tested in the remaining 40 patients.

## Results

Measurable regions of signal loss depicting TR jets were noted in 51/61 patients (84%), while 9/10 remaining patients had mild TR by echocardiography. With increasing severity of TR, jet area progressively increased from 6.7 ± 7.5 to 23 ± 14 to 38 ± 20 mm^2^, while the normalized SI decreased from 74 ± 26 to 39 ± 12 to 22 ± 11% (all p < 0.05). ROC analysis resulted in high AUC values in the derivation group and showed in the test group good accuracy (0.83 for both parameters), which was further improved by combining parameters.

## Conclusions

Severity of TR can be quantitatively assessed on short-axis CMR images in agreement with echocardiography. Pending future validation, this methodology may become part of the clinical CMR protocol without the need for acquiring additional images.

**Figure 1:**
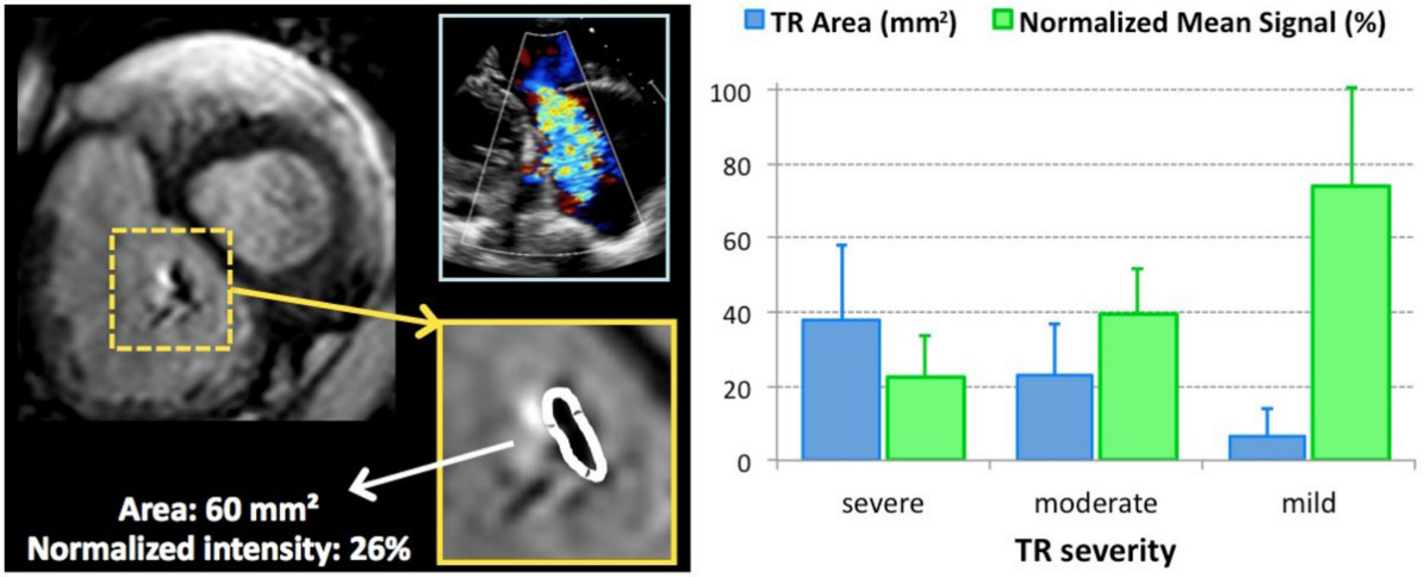
**Left: An example of a patient with severe TR assessed by both echocardiography (top right) and by CMR in short axis view, showing a signal loss in an area in the right atrium due to TR**. This TR jet area is shown without (left, zoomed insert) and with (bottom right) the manual tracing and measured values. Right: CMR-derived TR assessment by jet area and normalized signal intensity. With increasing severity of the TR, the area increased and the normalized signal intensity decreased (all p < 0.05).

**Figure 2:**
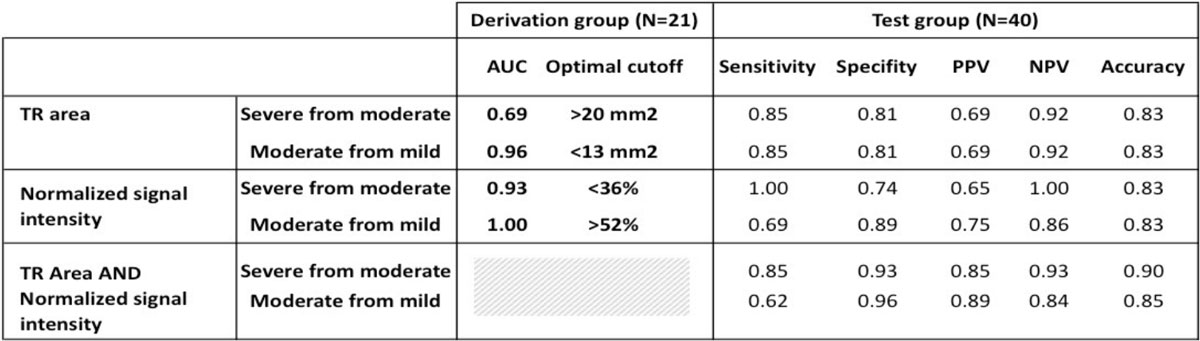
**Diagnostic accuracy of CMR-derived parameters of TR severity: area under ROC curves (AUC) and optimal cutoff values obtained in a derivation group of 21 patients (middle section), and the sensitivity, specificity, positive and negative predictive values (PPV, NPV) obtained by prospectively testing these cutoffs in an independent test group of 40 patients (right-hand section)**.

